# Resource utilisation and direct costs in patients with recently diagnosed fibromyalgia who are offered one of three different interventions in a randomised pragmatic trial

**DOI:** 10.1007/s10067-015-3067-y

**Published:** 2015-09-26

**Authors:** Yvonne van Eijk-Hustings, Mariëlle Kroese, An Creemers, Robert Landewé, Annelies Boonen

**Affiliations:** Department of Patient and Care, Maastricht University Medical Centre, PO box 5800, 6202 AZ Maastricht, The Netherlands; CAPHRI, School for Public Health and Primary Care, Maastricht University, Maastricht, The Netherlands; Department of Biostatistics, Hasselt University, Hasselt, Belgium; Department of Internal Medicine, Division of Rheumatology, Academic Medical Centre University of Amsterdam, Atrium Medical Centre Heerlen, Heerlen, The Netherlands; Department of Internal Medicine, Division of Rheumatology, Maastricht University Medical Centre, Maastricht, The Netherlands

**Keywords:** Fibromyalgia, Health care costs, Health resources, Intervention, Utilisation

## Abstract

The purpose of this study is to understand the course of costs over a 2-year period in a cohort of recently diagnosed fibromyalgia (FM) patients receiving different treatment strategies. Following the diagnosis, patients were randomly assigned to a multidisciplinary programme (MD), aerobic exercise (AE) or usual care (UC) without being aware of alternative interventions. Time between diagnosis and start of treatment varied between patients. Resource utilisation, health care costs and costs for patients and families were collected through cost diaries. Mixed linear model analyses (MLM) examined the course of costs over time. Linear regression was used to explore predictors of health care costs in the post-intervention period. Two hundred three participants, 90 % women, mean (SD) age 41.7 (9.8) years, were included in the cohort. Intervention costs per patient varied from €864 to 1392 for MD and were €121 for AE. Health care costs (excluding intervention costs) decreased after diagnosis, but before the intervention in each group, and increased again afterwards to the level close to the diagnostic phase. In contrast, patient and family costs slightly increased over time in all groups without initial decrease immediately after diagnosis. Annualised health care costs post-intervention varied between €1872 and 2310 per patient and were predicted by worse functioning and high health care costs at diagnosis. In patients with FM, health care costs decreased following the diagnosis by a rheumatologist. Offering patients a specific intervention after diagnosis incurred substantial costs while having only marginal effects on costs.

## Introduction

Fibromyalgia (FM) is characterised by chronic widespread musculoskeletal pain, often accompanied by other clinical manifestations such as fatigue and stiffness, but also cognitive dysfunction or mood disorders [1, 2]. FM typically affects women in working age. The prevalence of FM as reported among adults in Europe and in the USA varies between 2 and 4 % [3, 4]. While the aetiology of FM is unknown, the impact for the patient is high in terms of physical and mental suffering [5]. Moreover, FM is associated with substantial health resource utilisation and productivity loss, resulting in considerable societal cost-of-illness [3, 6–8]. In the literature, the average health care cost of FM varies from €1300–8300 per patient and is comparable or even higher than of rheumatoid arthritis (RA) or ankylosing spondylitis (AS) [9, 10]. When taking into account the prevalence of the diseases, however, the societal burden of FM is higher than that of RA or AS [11].

Various interventions have been studied, among which multi-modal non-pharmacological programmes as well as pharmacological therapies. However, the effectiveness of these interventions showed conflicting results [12, 13]. In the absence of substantial clinical effects, the cost-effectiveness of interventions in FM is disappointing [14–16]. The importance of a prompt (and earlier) diagnosis and of immediate (intensive) intervention are increasingly discussed as potential opportunities to prevent the development of persistent pain and long-term dysfunction and therefore might increase effectiveness of interventions [13, 17, 18]. Notwithstanding, only one randomised controlled trial (RCT) in FM patients showed that better treatment response was found in patients with a shorter disease duration [19].

Also, beneficial effects of a diagnosis in itself have been suggested but results of research, all conducted in claims databases, are conflicting; two studies showed that a diagnosis of FM increases costs, primarily attributable to an increased use of medication [20, 21], while two other studies suggested a reduction in costs attributable to a decrease in the number of visits to health care providers [22, 23]. Of interest, the first two studies were North-American and the latter two European.

The clinical burden of FM and the gaps in available evidence-based treatment recommendations [24, 25] justify a continued quest for innovations aimed at improving the outcomes. Such innovative approaches should first of all show effectiveness on health outcomes. In the absence of health improvements, reductions in cost-of-illness of FM through care innovations can be another reason to adopt health care innovations.

Previous analyses of a pragmatic trial among patients with recently diagnosed FM patients showed no consistent differences in relation to health outcomes between those that received a partially individualised multidisciplinary intervention with aftercare (MD), those receiving aerobic exercise (AE) and those receiving care as usual (UC) [26]. The present research aims to provide insight in resource utilisation and costs over 2 years in patients with FM after diagnosis and to understand whether the additional costs of specific interventions result in lower resource utilisation and costs.

## Patients and methods

A two-year cost-analyses of data of an observational study in which a pragmatic trial was embedded.

### Participants

A cohort of 203 consecutive patients that were recently (<3 months) diagnosed with FM according to the American College of Rheumatology criteria [1] at one of the Rheumatology Departments of three Medical Centres in the South of the Netherlands (Maastricht University Medical Centre (MUMC), Orbis Medical Centre, Sittard, and Atrium Medical Centre, Heerlen) were asked to participate in a study, as they were told, on the natural course of FM. Patients that consented were randomised to MD (*n* = 108), AE (*n* = 47) or UC (*n* = 48). Patients assigned to MD or AE were asked again to consent to participate in the proposed intervention without being aware of the alternative intervention. The study was approved by the Medical Ethical Committees of the participating medical centres. The precise procedures in this pragmatic trial, registration number ISRCTN32542621, have been published elsewhere [26].

### Interventions

The MD intervention was a two-phased group programme of 1 year. Phase l consisted of a 12-week course (three half days per week) with two therapy sessions of 1.5 h per day. Sociotherapy and physiotherapy were given twice per week; psychotherapy and creative arts therapy were given once per week. Phase II was an aftercare programme that was provided over the course of the remaining year and consisted of five group meetings. In addition, a maximum of seven individual therapy sessions with one of the therapists could be scheduled if considered necessary by the therapist and/or the patient. The AE intervention was a 12-week group course given twice a week by a trained physiotherapist in a community gym, following recommendations for exercise [27]. The UC group received ‘care as usual’ that comprised at least individualised education about FM and lifestyle advice by a rheumatologist or a specialised rheumatology nurse, but could also include referral to other interventions such as physiotherapy, or additional counselling by the rheumatology nurse.

### Baseline variables

Demographic characteristics (age, gender, education, work status) and health status were assessed by means of patient-reported questionnaires at entry in the observational study. Health status comprised symptom duration, and the impact of FM, measured by the Fibromyalgia Impact Questionnaire (FIQ), that consists of 10 items on health in the past week; physical functioning, numbers of days feel good, number of days missed work, interference of symptoms with ability to activities, pain, fatigue, unrefreshed sleep, stiffness, anxiety and depression [28]. Each item-score was standardised on a 0–10 scale after which a FIQ-total score (0–100) was calculated [28].

### Cost questionnaires and cost valuation

Self-reported FM-related health care resource use and costs for patients and their families served as a basis for the cost analysis and were collected by means of 12 cost diaries over a total study period of 2 years. In each diary, patients had to indicate the number of visits to general practitioners (GPs), medical specialists, physiotherapists and other paramedical therapists such as psychologists; the prescribed medication taken; the kind of assistive devices purchased; and the number of hours professional home help per week received during the 2 months prior to measurement [29]. Next, patients had to indicate the frequency with which they had participated in different types of health activities; the number and type of over the counter drugs that were purchased; the number of hours help from spouses, other relatives or paid household help per week received; and the number of prepared meals used, during the 2 months prior to measurement [29].

Health care costs for each category were calculated by multiplying the number of each resource used with its unit cost, derived from the Dutch Cost Manual or the Pharmacotherapeutic compass. If true costs were not available, market prices or tariffs were used [30–32]. Patient and family costs were calculated by either multiplying the number of resources used by costs per unit of the service, e.g. shadow prices in the case of informal home care, or using the price of the aid/service as stated by the patient [32]. Finally, total health care costs and total patient and family costs were summed. Costs for travel per patient were included in the total costs for visits to each provider or professional. Total direct costs were the sum of health care costs and patient and family costs.

### Intervention costs

The time input from all health care providers in the MD or AE group sessions were the basis for the calculation of the costs of the interventions. Total costs were calculated by summing the product of each hour of work by the gross salary per hour for each professional, augmented with 39 % charges for social security. As recommended, 45 % charges for overhead, including for use of accommodation, were added [32]. Finally, the total calculated costs for MD or AE were divided by the mean number of participants per group, resulting in a price per person per programme. The full intervention costs were considered for every person that started the interventions, even if patients did not complete the programmes. Costs for travel expenses to the sessions per patient were again added to the total intervention costs. Unit costs for wages and travel were indexed using the Dutch consumer price index rate for the value in 2010 (Centraal Bureau voor de Statistiek, Den Haag, Netherlands, www.cbs.nl) [32]. All cost and monetary values are presented in the Appendix (Table 4).

### Periods of interest

**Fig. 1:**
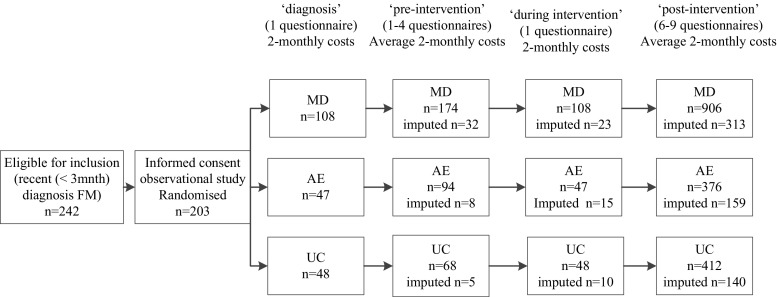
Study flowchart and measurements. *MD* multidisciplinary intervention (*N* = 108), *AE* aerobic exercise (*N* = 47), *UC* usual care (*N* = 48). *N* represents the number of patients, *n* represents the total number of questionnaires that were included in the analyses. In addition, the number of imputed questionnaires per period is presented

To understand the course of resource utilisation and costs per patient over time, we distinguished four clinical meaningful periods; the diagnostic phase representing the 2 months before referral to the rheumatologists and diagnosis of FM (diagnosis), the period after the diagnosis but before the intervention (pre-intervention), Phase I of MD, and AE (intervention) and the period after the intervention (post-intervention). As the start of the MD and AE programme varied in time among individual patients, the average number of questionnaires available for the period after the diagnosis but before the start of the MD or AE programmes varied from 1 to 4, and after the programmes from 6 to 9, while the main intervention period itself was always represented by one questionnaire. For the UC group, the number of questionnaires for each period was assigned after matching each subject randomly to a participant of the MD or AE group (see Figure 1).

### Statistical analysis

Intention-to-treat analyses, in which patients were analysed from the beginning according to the group in which they were randomised, were performed. Missing data occurred during the course of the study (12–30 %), and in order to achieve complete data, missing data were imputed using a non-parametric regression forest method [33].

First, mixed linear model analyses (MLM), with a random intercept, a random slope and an unstructured correlation structure, were used to assess differences between the intervention groups in the longitudinal course of health care costs and patient and family costs, with the baseline values of the dependent variables as covariates in the models.

Next, mean between group differences of the direct costs in- and excluding intervention costs over the total 2-year period were tested using a non-parametric bootstrap method to obtain 95 % confidence intervals.

Finally, to explore predictors of the health care costs in the period after the intervention a linear regression analysis on the square roots transformed costs was performed, entering as predictors age, symptom duration, educational level, FIQ-total score and health care costs in the diagnostic phase.

Data imputation was performed using R2.10.1, and all other analyses were performed using IBM Statistics SPSS version 20.0.

## Results

Baseline characteristics of the patients are presented in Table 1. The mean age was 41 years, and the majority of patients were female. Between 13 and 20 % had a high educational level, and about half of the patients were employed.

**Table 1:** Characteristics of the patients at entry into the observational cohort

	MD (*n* = 108)	AE (*n* = 47)	UC (*n* = 48)
Age, mean (SD) years	41.5 (9.6)	41.0 (9.0)	42.9 (11.0)
Female, %	93.5	100	97.9
Duration FM-related symptoms, mean (SD) years	6.9 (6.2)	6.9 (6.1)	7.1 (6.4)
FIQ-total score (0–100), mean (SD)	64.5 (13.7)	60.0 (12.3)	55.4 (15.1)
Married or cohabiting, %	84.2	85.1	83.4
Educational level, %			
- Low	56.7	57.8	38.7
- Medium	30.8	33.3	40.9
- High	12.5	8.9	20.4
Work status, % employed	49.5	55.6	50.0

*MD* multidisciplinary intervention, *AE* aerobic exercise, *UC* usual care, *FIQ* fibromyalgia impact questionnaire

The detailed course of health care as well as patient and family *resource utilisation* in the three groups is presented in the Appendix (Table 5). In summary, visits to GPs, medical specialists, physiotherapists and other paramedical professionals all decreased in each group after the diagnostic phase and before the intervention. In the AE group, visits to medical specialists and other paramedical professionals further decreased during the intervention period whereas visits to the physiotherapist increased somewhat in the MD and UC group. The use of formal home help increased in the three groups to higher levels than in the diagnostic phase. After the intervention, visits to medical specialists, physiotherapists and other paramedical professionals increased again in each group but remained lower than in the diagnostic phase for GP and medical specialist visits, and use of medication, while becoming higher for visits to physiotherapists, paramedical professionals and use of formal home care. Patient and family costs increased in each group from the diagnostic phase onward, but the largest increase in costs was seen in attending health activities, and in using paid home help or informal care.

**Fig. 2:**
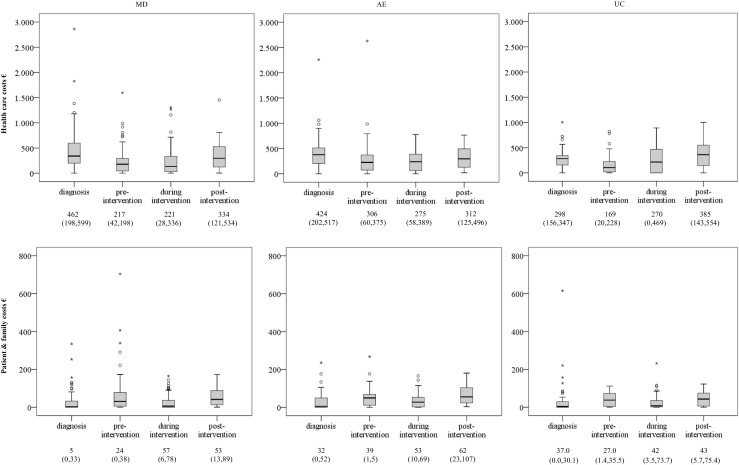
The course of health care costs and patient and family costs per 2 months, averaged over the period. The figure presents median, IQR, range and outliers. *MD* multidisciplinary intervention, *AE* aerobic exercise, *UC* usual care

*Health care costs*, excluding intervention costs, decreased statistically significantly after diagnosis but before the intervention in all groups (Figure 2). During the intervention period, costs in the AE group tended to decrease further and remained unchanged in the MD group but showed a statistically significant increase in the UC group. Post-intervention, health care costs increased again in all groups, although not statistically significant in the AE group. In the UC group, health care costs post-intervention were higher than in the diagnostic phase. MLM confirmed that time had a different influence on the course of health care costs in the three groups with statistically significant differences between the AE and UC group (*F* = 5.3, *p* = 0.01) (not presented), reflecting a sooner increase of costs in the UC group (after the initial similar decrease). Patient and family costs increased in the three groups. MLM indicated no statistical significance between group differences.

Patient and family costs increased in the three groups. MLM indicated no statistical significance between group differences.

While the changes in costs incurred by the intervention were marginal and not statistically significant, the intervention costs were substantial; for MD €864 per person in phase I and in phase II, the costs varied between €86 and 528 per person depending on the number of individual contacts. Costs for AE were €121 per person. The resulting total direct costs per person (with and without programme costs) over the total observation period after the diagnostic phase are presented in Table 2. Total direct costs, including the intervention costs, were highest in MD. However, between group differences were not statistically significant.

**Table 2:** Costs in € per patient over the total 2 years after the diagnostic phase

Costs				Group		
	UC	MD	AE	Mean difference MD vs UC (CI)^a^	Mean difference AE vs UC (CI)^a^	Mean difference MD vs AE (CI)^a^
	Mean, median (IQR)	Mean, median (IQR)	Mean, median (IQR)			
Health care costs	3800, 3625 (1681, 5788)	3510, 3151 (1204, 5294)	3594, 3337 (1990, 5103)	−290 (−1134, 605)	−206 (−1207, 806)	−84 (−855, 892)
Patient and family costs	452, 453 (100, 710)	565, 426 (147, 937)	678, 711 (258, 1030)	113 (−14, 250)	226 (60, 388)	−113 (−48, 270)
Total direct costs						
Excluding intervention costs	4252, 3973 (2054, 6488)	4075, 3766 (1872, 6167)	4272, 3725 (2127, 5788)	−177 (−1095, 767)	20 (−1067, 1158)	−197 (−815, 1106)
Including intervention costs	4252, 3973 (2054, 6488)	4740, 4510 (2248, 6651)	4321, 3725 (2127, 5788)	488 (−418, 1458)	69 (−1025, 1202)	419 (−1370, 479)

*MD* multidisciplinary intervention, *AE* aerobic exercise, *UC* usual care

^a^Mean difference: bootstrapped mean difference (95 % confidence interval)

Finally, the 2-monthly health care costs in the post-intervention period were predicted by a high impact of FM (FIQ) already at entry in the cohort and by higher health care costs before the intervention (see Table 3).

**Table 3:** Prediction of cost in the period after intervention

Predictors assessed at diagnosis (*n* = 203)	Unstandardised coefficients		Standardised coefficients	*p* value	95.0 % confidence interval (CI) for B	
	*B*	Standard error	Beta		Lower bound	Upper bound
(Constant)	6.7	3.7		0.07	−0.595	14.065
FIQ-total score	0.093	0.038	0.173	0.01	0.019	0.167
Health care costs	0.004	0.001	0.187	0.01	0.001	0.007
Age	0.080	0.054	0.104	0.14	−0.025	0.186
Duration symptoms	0.117	0.083	0.096	0.16	−0.046	0.281
Education	−0.861	0.760	−0.080	0.26	−2.361	0.638

*R* ^2^ 11.9 %; dependent variable: square root transformed health care costs post-treatment

*FIQ* fibromyalgia impact questionnaire

## Discussion

This article describes the course of resource utilisation and costs in patients that recently received a diagnosis of FM by the rheumatologist who offered them, soon thereafter, one of three interventions (MD, AE and UC) along a randomised pragmatic trial. While the costs of the MD and AE intervention were substantial, their influence on health care costs and patient and family costs over time was similar.

The major interest of the paper can be found in the course of health care utilisation and costs over time which decreased immediately after the diagnoses, before the MD or AE intervention was started. Since we previously failed to show any effects of the interventions on different aspects of health, including the 5-dimensional EuroQol (EQ-5D) [34] and FIQ as reported in our previous publication [26], a cost-minimisation study of the pragmatic trial could have been considered. However, a classical cost-minimisation study starts from the intervention onward and would have ignored the course of the costs incurred during diagnosis and therefore would have failed to show the large decrease in health care costs after the diagnostic phase and before the start of a specific intervention. We considered this observation remarkable and decided to present the evolution of costs from entry into the cohort and not from the start of the intervention only.

The higher health care costs around the period of diagnosis likely reflect the high needs of patients to find help for their complaints. It is understandable that GPs first explore several diagnostic and treatment options in such periods and next refer patients, if complaints persist, to a rheumatologist for confirmation of the diagnosis and for a better treatment plan. In addition, the diagnosis itself includes several consultations and therefore costs are higher in this period as in FM, follow-up visits are limited. The decrease in costs after the diagnosis could partially be attributed to regression to the mean. Notwithstanding, it cannot be excluded that also the diagnosis itself reassures patients and reduces resource utilisation in the period short after diagnosis and before the start of the MD or AE programme [23, 35].

The increase in patient and family costs, reflecting increased participation in health activities, and increased use of paid help and informal care over time is not surprising. In the usual care setting, patients receive information and education from the rheumatologist, the rheumatology nurse or the GP and they are encouraged to implement sports, to pace their tasks and activities and ask for support from relatives and friends [36, 37]. Our findings confirm adherence to such lifestyle advices.

Our results have far reaching consequences for clinical practice which is a strength of our study, as it suggests that care as usual in the Netherlands is as good and substantially cheaper compared to more complex interventions and might be appropriate for many patients.

However, some limitations need to be addressed. First, we have used data from a cohort of patients participating in a trial and missing data occurred within the cohort. Missing data were carefully explored and were imputed. Of course, some level of uncertainty cannot be excluded but the advanced method that was used for the data imputation limits errors and contributes to valid data. Also, we did not include indirect costs in our study. Only 50 % of the patients were employed, and this small sample would limit the possibility for a reliable assessment of the course of costs due to productivity loss. Further, our study comprised a cohort of recently diagnosed patients but in the cohort, there was not necessarily a recent onset of FM. Likely, patients had been managed by their GPs during a longer period. Well-trained GPs in the Netherlands can diagnose and manage FM appropriately [38], but it is recognised that some GPs avoid mentioning the diagnosis FM for several reasons. Apparently, a number of patients raise concerns about the exact diagnosis and about the treatment options and these patients can be referred to a rheumatologist. Finally, with regard to the generalisability, it should be emphasised that patients were referred by GPs and therefore might present the somewhat more severe spectrum of the disease. In addition, patients agreeing to participate in the active interventions might have been already more motivated. The central role of the GP in referral, the attitude of persons towards self-management and the content of usual care might be different across countries and cultures and, therefore, it cannot be excluded that this intervention might be more (cost-) effective in other countries of health care settings.

In summary, our results show that after diagnosing FM, the use of health care resources decreases, and the slight increase afterwards is largely independent of the interventions offered. Given the absence of beneficial health effects but additional high intervention costs of MD and AE, such interventions cannot be recommended to all patients with FM. Notwithstanding, we cannot exclude that a subgroup of patients might have benefit from MD or AE; an appropriate selection of patients for interventions may result in larger effects and may contribute to cost-effectiveness. Future research should focus on improved selection of patients for specific health care innovations.

## Appendix

**Table 4:** Cost categories, units and sources of estimate, costs in Euro (€) per unit (including travelling expenses)

Cost category	Source of estimate	Costs per unit, € (2010)
Interventions		
Multidisciplinary programme		
Phase I: programme	Hakkaart-van Roijen, calculated [32]	863.94/programme
Phase II: aftercare	Hakkaart-van Roijen, calculated [32]	85.79/programme
Individual sociotherapy	Hakkaart-van Roijen, calculated [32]	50.23/contact
Individual creative therapy	Hakkaart-van Roijen, calculated [32]	50.23/contact
Individual psychotherapy	Hakkaart-van Roijen, calculated [32]	63.17/contact
Aerobic exercise	Hakkaart-van Roijen, calculated [32]	120.47/programme
Health care costs		
General practitioner		
Practice	Hakkaart-van Roijen [32]	31.58/contact
Home visits	Hakkaart-van Roijen [32]	43.56/contact
Telephone	Hakkaart-van Roijen [32]	14.18/contact
Out of hours services, practice	Hakkaart-van Roijen [32]	67.42/contact
out of hours services, telephone	Hakkaart-van Roijen [32]	26.26/contact
Medical specialist outpatient service		
Academic	Hakkaart-van Roijen [32]	135.08/contact
Not academic	Hakkaart-van Roijen [32]	69.23/contact
Paramedical professionals		
Physiotherapy	Hakkaart-van Roijen [32]	39.91/contact
Cesar or Mensendieck therapy	Hakkaart-van Roijen [32]	38.90/contact
Occupational therapy	Hakkaart-van Roijen [32]	38.90/contact
Social work	Hakkaart-van Roijen [32]	69.29/contact
Activity therapy	Hakkaart-van Roijen, calculated [32]^a^	19.90/contact
Psychotherapy	Hakkaart-van Roijen [32]	82.96/contact
Other	Patient-reported costs	Various/contact
Prescribed medications	Pharmacotherapeutic compass 2007 [30]	Various/DDD^b^
Assistive devices	Estimated market price^c^	Various
Professional home help	Hours [32]	24.31/h
Patient and family costs		
Health activities	Patient-reported costs	Various
Over the counter medications	Patient-reported costs	Various
Prepared meals	Estimated market price	7.26/meal
Paid home help	Patient-reported costs	Various/h
Informal care	Hakkaart-van Roijen [32]	12.66/h

^a^Group session, based on costs for social work: costs individual session/4

^b^DDD: daily defined dosage

^c^Various websites: www.thuiszorgwinkel.nl, www.groenekruisdomicurazorg.nl, www.medireva.nl

**Table 5:** Health care and patient and family resource use per patient in each of the observation periods

MD *n* = 108		Diagnosis	Pre-intervention^a^	During intervention	Post-intervention^a^
AE *n* = 47					
UC *n* = 48					
Health care					
GP	MD	2.3 (0.0, 3.0)	1.0 (0.0, 1.3)	1.2 (0.0, 1.8)	1.1 (0.3, 1.6)
	AE	3.3 (0.0, 2.0)	1.0 (0.0, 1.5)	2.1 (0.0, 2.5)	1.2 (0.4, 1.7)
	UC	1.4 (0.0, 2.0)	0.4 (0.0, 0.5)	0.5 (0.0, 0.5)	0.8 (0.3, 1.1)
Medical specialists	MD	2.0 (1.0, 2.0)	0.3 (0.0, 0.4)	0.2 (0.0, 0.2)	0.5 (0.0, 1.0)
	AE	1.9 (1.0, 2.0)	0.4 (0.0, 0.5)	0.2 (0.0, 0.2)	0.8 (0.1, 1.6)
	UC	1.6 (1.0, 2.0)	0.4 (0.0, 0.5)	0.4 (0.0, 0.6)	0.5 (0.0, 0.9)
Physiotherapist	MD	2.7 (0.0, 4.0)	1.8 (0.0, 2.0)	2.3 (0.0, 2.3)	3.2 (0.3, 5.3)
	AE	2.9 (0.0, 4.0)	2.2 (0.0, 4.0)	2.2 (0.0, 1.3)	2.3 (1.1, 2.1)
	UC	1.0 (0.0, 3.0)	1.3 (0.0, 0.9)	2.6 (0.0, 2.6)	2.7 (0.2, 2.9)
Other paramedical professionals	MD	1.1 (0.0, 0.0)	0.6 (0.0, 0.6)	0.8 (0.0, 0.9)	1.9 (0.0, 4.1)
	AE	1.1 (0.0, 1.0)	1.2 (0.0, 1.8)	1.0 (0.0, 1.7)	2.0 (0.2, 3.5)
	UC	0.6 (0.0, 0.0)	1.2 (0.0, 1.0)	1.2 (0.0, 2.6)	1.6 (0.0, 3.1)
Formal home help	MD	0.1 (0.0, 0.0)	0.2 (0.0, 0.0)	0.4 (0.0, 0.0)	0.4 (0.0, 0.0)
	AE	0.0 (0.0, 0.0)	0.0 (0.0, 0.0)	1.0 (0.0, 0.0)	1.1 (0.0, 0.0)
	UC	0.1 (0.0, 0.0)	0.1 (0.0, 0.0)	0.6 (0.0, 0.0)	0.5 (0.0, 0.0)
Use prescribed drugs (% yes)	MD	73.1	58.6	60.0	52.3
	AE	59.6	60.5	56.3	53.0
	UC	66.7	41.3	39.5	48.5
Use assistive devices (% yes)	MD	5.6	7.2	3.5	3.4
	AE	2.1	5.8	6.3	5.5
	UC	6.3	3.2	5.3	8.8
Patient and family					
Paid home help	MD	0.1 (0.0, 0.0)	0.1 (0.0, 0.0)	0.0 (0.0, 0.0)	0.3 (0.0, 0.5)
	AE	0.2 (0.0, 0.0)	0.2 (0.0, 0.0)	0.4 (0.0, 0.8)	0.8 (0.0, 1.7)
	UC	0.0 (0.0, 0.0)	0.2 (0.0, 0.0)	0.1 (0.0, 0.0)	0.1 (0.0, 0.2)
Informal care	MD	0.4 (0.0, 0.0)	0.6 (0.0, 0.0)	1.6 (0.0, 1.5)	0.9 (0.0, 1.1)
	AE	0.1 (0.0, 0.0)	0.7 (0.0, 0.5)	1.2 (0.0, 1.3)	1.1 (0.0, 1.6)
	UC	0.3 (0.0, 0.0)	0.3 (0.0, 0.0)	0.6 (0.0, 0.4)	0.6 (0.0, 0.8)
Prepared meals	MD	0.0 (0.0, 0.0)	0.0 (0.0, 0.0)	0.1 (0.0, 0.0)	0.0 (0.0, 0.0)
	AE	0.0 (0.0, 0.0)	0.0 (0.0, 0.0)	0.0 (0.0, 0.1)	0.0 (0.0, 0.0)
	UC	0.0 (0.0, 0.0)	0.0 (0.0, 0.0)	0.0 (0.0, 0.0)	0.0 (0.0, 0.0)
Health activities (% yes)	MD	24.1	21.7	29.4	33.1
	AE	27.7	36.0	34.4	35.0
	UC	16.7	28.6	39.5	18.8
Use over the counter medication (% yes)	MD	53.7	47.4	42.4	45.0
	AE	40.4	37.2	31.3	31.3
	UC	56.3	57.1	68.4	58.8

Resource use per 2 months; mean (IQR) or percentages of resource use

*MD* multidisciplinary intervention, *AE* aerobic exercise, *UC* usual care

^a^Average 2-monthly resource use over the period
